# Dysregulation of objectively assessed 24-hour motor activity patterns as a potential marker for bipolar I disorder: results of a community-based family study

**DOI:** 10.1038/tp.2017.136

**Published:** 2017-08-22

**Authors:** H Shou, L Cui, I Hickie, D Lameira, F Lamers, J Zhang, C Crainiceanu, V Zipunnikov, K R Merikangas

**Affiliations:** 1 Department of Biostatistics, Epidemiology, and Informatics, University of Pennsylvania Perelman School of Medicine, Philadelphia, PA, USA; 2 Genetic Epidemiology Research Branch, Intramural Research Program, National Institute of Mental Health, Porter Neuroscience Research Center, Bethesda, MD, USA; 3 Brain and Mind Institute, University of Sydney, Sydney, NSW, Australia; 4 Department of Psychology, George Mason University, Fairfax, VA, USA; 5 Department of Psychiatry, EMGO Institute for Health and Care Research, VU University Medical Centre, Amsterdam, The Netherlands; 6 Department of Psychiatry, Chinese University of Hong Kong, Hong Kong, PRC; 7 Department of Biostatistics, Johns Hopkins Bloomberg School of Public Health, Baltimore, MD, USA

## Abstract

There has been a growing number of studies that have employed actigraphy to investigate differences in motor activity in mood disorders. In general, these studies have shown that people with bipolar disorders (BPDs) tend to exhibit greater variability and less daytime motor activity than controls. The goal of this study was to examine whether patterns of motor activity differ in euthymic individuals across the full range of mood disorder subtypes (Bipolar I (BPI), Bipolar II (BPII) and major depression (MDD)) compared with unaffected controls in a community-based family study of mood spectrum disorders. Minute-to-minute activity counts derived from actigraphy were collected over a 2-week period for each participant. Prospective assessments of the level, timing and day-to-day variability of physical activity measures were compared across diagnostic groups after controlling for a comprehensive list of potential confounding factors. After adjusting for the effects of age, sex, body mass index (BMI) and medication use, the BPI group had lower median activity intensity levels across the second half of the day and greater variability in the afternoon compared with controls. Those with a history of BPII had increased variability during the night time compared with controls, indicating poorer sleep quality. No differences were found in the average intensity, variability or timing of activity in comparisons between other mood disorder subgroups and controls. Findings confirm evidence from previous studies that BPI may be a manifestation of a rhythm disturbance that is most prominent during the second half of the day. The present study is the largest study to date that included the full range of mood disorder subgroups in a nonclinical sample that increases the generalizability of our findings to the general community. The manifestations of activity patterns outside of acute episodes add to the accumulating evidence that dysregulation of patterns of activity may constitute a potential biomarker for BPD.

## Introduction

Actigraphy, a reliable, non-invasive^1, 2^ and objective method to monitor 24-h patterns of motor activity over time^3^ has been increasingly used in both community and clinical studies of chronic diseases such as diabetes,^4^ heart disease^5^ and dementia.^6^ Actigraphy has also been used to study neuropsychiatric disorders, particularly affective disorders,^7, 8, 9^ and attention deficit hyperactivity disorders.^10, 11^ Most research on mood disorders has employed actigraphy as an index of sleep onset, offset or quality rather than as a direct measure of 24-h patterns of motor activity.^1, 12, 13, 14, 15, 16, 17^ There has been a growing number of studies of individuals with bipolar disorder (BPD),^18^, ^19^, ^20^, ^21^, ^22^, ^23^, ^24^, ^25^, ^26^, ^27^, ^28^ major depression (MDD)^7, 29^ and those at risk for BPD^14, 30, 31^ that have employed actigraphy to investigate differences in 24-h patterns of activity associated with mood disorders, as recently summarized by Scott et al. ^32^ In general, these studies have shown that people with BPD tend to exhibit greater variability and less daytime motor activity than controls. Increased variability in daily activity has also been found among various subgroups of people with MDD,^7, 22^ particularly the seasonal and atypical subtypes. Comparisons of differences in sleep and activity within^22, 33^ and between episodes of BPD and/or MDD^13, 25, 34, 35^ have shown that differences in activity patterns may constitute trait rather than state manifestations of mood disorders.

Aggregation of the data across these studies is complicated by the substantial differences in the goals, procedures, samples and statistical methods,^36^ particularly estimates of average and variability in daily activity.^7, 37, 38^ The majority of studies of mood disorders have been based on relatively small clinical samples of patients with BPD. Analytic methods have also been highly variable, and few studies have simultaneously estimated the three key measures of motor activity (for example, magnitude, timing and variability) while accounting for correlations of time-dependent activity levels across multiple days within the same subject.

In this paper, we examined the association between mood disorder subtypes and objectively measured motor activity in a large community-based family study, the National Institute of Mental Health Family Study of Affective Spectrum Disorders.^39^ The chief goal is to evaluate differences in the magnitude, timing and day-to-day variability of activity intensity derived from actigraphy among euthymic individuals with a history of mood disorders including Bipolar I (BPI), Bipolar II (BPII) and MDD as compared with unaffected controls.

## Materials and methods

### Sample and procedures

The samples were participants in the National Institute of Mental Health Family Study of Affective Spectrum Disorder, a large community-based controlled family study of probands assessed for the full range of mood disorders. Probands for the family study were recruited from a survey of the local community and enriched through volunteers and referrals from the National Institute of Health Clinical Center. The only inclusion criteria for this phenomenological family study were the ability to speak English, availability to participate in the study and consent to contact at least two living first-degree relatives. Among the enrolled probands, 73% had at least one first-degree adult relative with a diagnostic interview, and 71% of the first-degree relatives who were alive and could be located were enrolled in the study; of these relatives, 73% were directly interviewed and family history information was systematically collected from probands and interviewed relatives regarding a total of 1523 living and deceased adult first-degree relatives, yielding a total of 2082 first-degree relatives. The study was approved by the Combined Neuroscience IRB at the National Institute of Health. All participants provided written informed consent. More details of the family study methods are presented in Merikangas *et al.*^39^

The subsample included in this paper were 339 participants including 172 probands and 167 relatives who reside in the greater Washington, DC area and underwent evaluation at the NIH Clinical Center, where they had a comprehensive evaluation including physical examination, neuroimaging and neurocognitive testing. Body mass index (BMI) was calculated from height and weight that were directly measured by nurse clinicians. Lifetime and current medication use was assessed through direct interview with the participants. Sixteen percent of the participants, including ~40% of those with a history of BPI and MDD, were taking antidepressants, and ~25% of those with BPI were taking antimanic medications including anticonvulsants (*n*=3) and lithium (*n*=5) at the time of the Clinical Center visit. Global assessment of functioning was assessed for overall functioning level, including psychological, social and occupational/school functioning, but excluding physical or environmental limitations as part of the psychiatric interview.

Table 1 provides the demographic distribution, information on BMI and medication use that were included as covariates in the analyses due to their potentially confounding effect on motor activity.^40, 41^ The mean age was 41.9 years, with a range from 10 to 84, and 60% of the sample was female. There was a gradient of severity of lifetime global functioning across diagnostic subgroups from controls with no evidence of functional impairment, those with a history of MDD and BPII exhibiting intermediate levels of impairment and the BPI group with the greatest level of lifetime functional impairment. None of the participants was suffering from an acute severe episode of a mood disorder because the goal of the study was to assess people outside of acute episodes. Controls were required to have no lifetime history of mood or other mental disorders.

### Measures

#### Diagnostic assessments

All of the participants in this study were directly interviewed by experienced clinical interviewers using a comprehensive semistructured diagnostic interview that collected information on the symptoms, duration, severity, impairment and recurrence of mood disorders and other major classes of mental disorders defined by Diagnostic and Statistical Manual for Mental Disorders, IV^th^ Edition (DSM-IV) criteria. Mood disorder subgroup diagnoses were based on all available information reviewed by a team of experienced clinicians (psychologists or psychiatrists) using a best estimate procedure.^39^

#### Activity monitoring

Actigraphy (Actiwatch Spectrum, Philips Respironics, Murrysville, PA, USA) was used to produce the minute-by-minute activity counts for the participants to monitor their sleep/activity cycles. Actiwatch is a device widely used to document sleep/wake patterns in both general population and patients with either sleep or psychiatric disorders.^42^ Sleep/activity parameters derived from the actiwatch have been shown to differentiate people with BPD from controls.^16^ Activity counts quantify the intensity of movement during a 1-min epoch by summarizing the voltage signals recorded by the accelerometer during movement.

The participants were instructed to wear the device on their non-dominant wrist for 2 weeks. The device includes buttons for participants to document important events, such as removing the watch, going to sleep, waking up, and so on. Participants also completed self-ratings of mood and anxiety states at the time of actigraphy assessments.

### Statistical methods

All actiwatches were manually reviewed to identify missing data according to the following criteria: (1) non-wear time as identified by the Actiwatch software and validated manually by experienced staff members; (2) incomplete data collections on the first and last days of the study by design; or (3) intermittent missingness potentially due to the insensitivity of the device or manual exclusion of outlier values by an experienced staff member. The average proportion of missing data within a day for the study participants is 18.4%, and the average length of consective missing interval is ~2 h. After removing the first and last days of the study, we imputed the missing values by replacing the missing activity counts at a specific time point using the average values from other days at the same time from the same subjects.^43^ Imputation enabled us to incorporate the majority of the information from the data.

For each of the 339 subjects, we analyzed daily activity profiles represented by 1440 minute-level activity counts. In order to ensure the symmetry of the distributions of activity counts, we applied a log (count+1) transformation. Transformed activity counts were then averaged within the following time intervals: 00:00–06:00, 06:00–12:00, 12:00–16:00, 16:00–20:00 and 20:00–00:00 hours. Next, we separately modeled associations between averaged activity at each time interval and mood disorders using the generalized estimating equation (GEE) approach^44^ while adjusting for potential confounders including age, gender, BMI, medication and a weekend indicator. The GEE models account for correlations of activity levels across multiple days within the same subject. Secondly, to examine the day-to-day variability in the activity measures, we employed a two-stage approach by first calculating the standard daviation of the interval-specific average log activity counts across days for each participant and each time interval. We then fit linear regression models of standard deviation. on age, gender, BMI, mood disorder subgroup, medication use and weekend versus weekday (1 versus 0) within each of the five intervals.

In order to test familial effects, we conducted stratified analyses of the data by proband and relative status and then included a parameter for familial clustering in GEE models, and the results were essentially the same. The lack of a familial effect is most likely attributable to the small number of families with multiple relatives who participated in the direct clinical component of this study.

## Results

**Figure 1:**
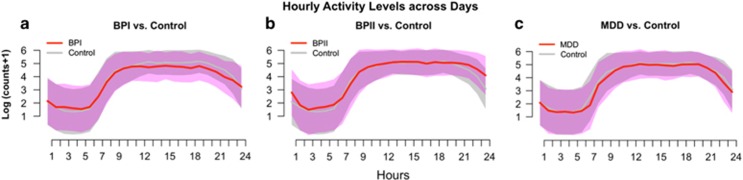
The distribution of hourly log activity counts across days comparing participants with different mood disorder subtypes (red curve and pink band) to those of Controls (gray curve and gray band). For each panel, the curves represent the median activity counts for participants within the specific group. The width of the band around each curves represents the average day-to-day variability (s.d.'s) calculated for each subgroup. (**a**) Activity profiles of individuals with bipolar I disorder (BPI) compared with controls. Panel **a** shows that those with BPI have lower activity between noon to evening and the wider pink than the gray band indicates greater average day-to-day variation among those with BPI. The median activity levels in those with Bipolar II Disorder (BPII; **b**) and major depressive disorder (MDD; **c**) are similar to those of controls.

Figure 1 shows box plots of the hourly averages of log activity counts across all participants and days for the BPI, BPII and MDD groups as compared with the controls. The BPI group had lower median activity intensity levels and higher variation in the hourly activity counts in the period from the afternoon until midnight than controls. Comparisons between the BPII and MDD subgroups with controls yielded no significant differences.

### Differential average activity levels

Table 2 presents estimates from the GEE models for the log-transformed activity counts across five time epochs for the mood disorder subgroups after adjustment for age, gender, BMI, weekend versus weekday, and medication use. Motor activity was associated with age and weekday versus weekend for most of the time epochs. For example, older participants were less active in the afternoon, evening and nighttime (12:00–06:00 hours) than the younger age groups, but also tended to have a higher average activity level (not statistically significant), probably attributable to an earlier average wake-up time. There was less activity on weekends during the morning, evening and the sleeping period, but greater activity between 20:00 and 00:00 hours (not statistically significant), most likely due to increased nighttime activities and possibly delayed bedtime. Females had higher levels of activity between 12:00 and 20:00 hours. Higher levels of BMI were associated with lower levels of activity from 1200 to 2000 hours, but more activity during the sleep period, suggesting poorer sleep quality. As shown in Figure 1, the BPI subgroup had significantly lower activity (12:00–00:00 hours) than the control group (that is, 0.36–0.51 lower in log activity counts, or equivalently about 60% of the activity intensity compared with controls), while other mood disorder subgroups did not differ significantly from controls. Concurrent psychotropic medication use was not associated with differential levels of activity in our sample.

### Variability

Table 3 shows that after adjusting for confounders, the BPI subgroup exhibited larger day-to-day variability during the afternoon period (12:00–16:00 hours), while those with BPII had greater variability during the evening period (00:00–06:00 hours) as compared with controls. There were no differences in the day-to-day variability between the MDD group and controls.

## Discussion

The greater variability and reduced daytime activity among people with BPI disorder are consistent with accumulating evidence that BPD may be a manifestation of a circadian rhythm disturbance.^2, 14, 22, 32, 45, 46^ In fact, our finding of greater differences between BPD and controls in daytime rather than nighttime activity suggests that these patterns extend beyond disrupted patterns of sleep, which has been the primary focus of earlier studies.^13, 16^ The manifestations of activity patterns outside of acute episodes in both probands and their relatives^19, 33, 34^ combined with evidence from studies of people with increased vulnerability to BPD^14, 30, 47^ or genetic liability to BPD^28, 48^ provide evidence that dysregulation of activity may constitute a potential endophenotype for BPD.^49^

The specificity of these findings with respect to BPI compared with BPII and MDD indicates that these differences in activity patterns may be attributable to the correlates of mania rather than to the depression component of BPD, thereby confirming evidence from clinical^50, 51^ and family^39^ studies that discriminate BPI from BPII and MDD. Although most prior studies of potential biomarkers or endophenotypes of BPD have considered the full spectrum of BPD as a single entity, these findings imply that this heterogeneity should be considered in future research, particularly through extension beyond clinical samples.

The systematic comparison of weekend and weekday activity in mood disorders, which would have been missed by cross-day averages, has not been included in prior studies. Differential weekday–weekend patterns of level of activity induced by social structure, known as 'social jetlag',^52^ have been associated with several adverse endocrine, behavioral and cardiovascular profiles.^53^ Of interest, our concomitant modeling of mood with activity demonstrated a diminution of low mood on weekends that may reflect greater sleep duration, as well as a shift in peak activity to later in the day. Future studies of the significance of weekend–weekday differences in those with mood disorders are clearly indicated.

The present study is the largest study to date that included the full range of mood disorder subgroups in a nonclinical sample that increases the generalizability of our findings to the general community. Several methodological features of this study also have implications for future research that seeks to provide insight into the biological, environmental and social factors underlying activity patterns associated with BPD. First, we demonstrate the importance of consideration of demographic correlates including age and gender,^54, 55, 56, 57, 58^ and health correlates (BMI) that may confound the associations between mood disorders and objectively assessed activity. We also show differential timing of patterns of activity in males and females, such that females had higher average activity levels than males later in the day (12:00–20:00 hours), demonstrating the importance of timing of activity that may be obscured by inspection of average activity across the day.^55, 56^ Although we did not have sufficient power to test interactions among these covariates, our findings did indicate that BMI is related to lower activity during the daytime, but to greater activity during the nighttime.

This paper also highlights the importance of standardization of procedures,^7, 38, 59, 60^ and statistical methods^21, 61^ that are critical for interpretation of our findings in the context of prior work. Novel features of these analyses include the following: (1) incorporation of time-varying associations of activity and disorders by splitting each day into five time intervals; (2) incorporating the day-to-day correlations from repeated observations via GEE modeling; and (3) modeling of variability and its interaction with time as a novel marker that distinguished between mood disorder subgroups. By splitting the 24-h period into five time intervals and modeling the mean and standard variation separately in each using GEE and linear models, we attempted to create a more flexible and more sensitive modeling framework. The time-varying associations of activity have often been neglected in prior analyses that relied on averages without their temporal manifestations that may mask associations with particular time periods of the day. In order to address these methodological issues and to increase the sample size of people with BPD and actigraphy, we have established a large collaborative international consortium (Motor Activity Research Collaborative Network-*mMARCH*) to coordinate research on motor activity in mood disorders using common procedures and analytic methods.

There are also limitations of our study that should be considered in interpreting the findings. First, we assume that the daily activity profiles by participant and day are aligned on a common 24-h time domain. We are now employing methods to account for variability in the length of the circadian period to normalize the daily activity profiles. Second, although the study was enriched for mood disorders, these findings may not apply to more severely ill people with BPD or psychotic affective syndromes. However, our findings align with those of several studies that have examined activity patterns in clinical samples of people with mood disorders.^32^ Third, although we controlled for medication use and no effect was found across the five time periods, the lack of effect may have been attributable to low power to detect a difference. Fourth, although our ultimate aim is to examine whether motor activity patterns may be an endophenotype for BPD, we do not yet have sufficient power to test this concept because of the relatively small family size in this sample to date. Efforts are now underway to record activity in relatives who do not reside in the local area. Fifth, the findings remain a cross-sectional snapshot of activity that may not extend across time and seasons. We are now repeating these assessments at multiple time points throughout the year to investigate seasonal influences and other fluctuations in activity patterns.

These findings have both etiologic and therapeutic heuristic implications. The differences in rhythms of activity in BPD support growing evidence for dysregulation of circadian biology and its environmental inputs in BPD.^46^ Advances in characterizing the molecular biology of circadian systems of sleep and activity and their environmental determinants^62^ will enhance insight into both the genetic and contextual factors involved in potential dysregulation of these systems in BPD. In fact, the rapid growth in knowledge regarding the neural and metabolic correlates of misalignment between intrinsic biologic systems and their behavioral correlates across the whole 24-h light–dark cycle, and their major health impact^63^ has led to the development of circadian medicine as a new frontier in medicine.^64^ Such progress is likely to have major implications for the treatment and prevention of episodes of BPD. Greater insight into mechanisms will inform interventions such as rhythm^65, 66^ or light therapy^17^ that seek to stabilize daily rhythms, anticipate recurrence and possibly to prevent onset and recurrence in vulnerable individuals.^35, 67, 68^

## Disclaimer

The views and opinions expressed in this article are those of the authors and should not be construed to represent the views of any of the sponsoring organizations, agencies or US Government.

## Figures and Tables

**Table 1:** Demographic and health characteristics of study sample by mood disorder subgroups

	Mood disorder subgroup					P
	*Bipolar I*	*Bipolar II*	*Major depression*	*Controls*	*Total*	
*N* (%)	33 (9.73%)	31 (9.14%)	52 (15.34%)	223 (65.78%)	339	
Age (mean, s.d.)	39.5 (14.1)	38.4 (17.1)	44.4 (18.6)	42.2 (20.9)	41.9 (19.7)	0.50
Range (years old)	13–65	13–66	12–78	10–84	10–84	
						
*Gender (%)*						
Male	36.4%	35.5%	28.9%	44.4%	40.4%	0.18
Female	63.6%	64.5%	71.1%	55.6%	59.6%	
BMI (mean, s.d.)	29.31 (9.19)	27.50 (6.40)	26.91 (6.48)	26.95 (6.96)	27.23 (7.08)	0.39
Antidepressant use (%)	39.4%	12.9%	44.2%	7.2%	16.5%	<0.001
GAF (mean, s.d.)	58.39 (9.06)	64.39 (6.91)	62.98 (8.75)	73.17 (7.53)	69.20 (9.83)	<0.001

Abbreviations: BMI, body mass index; GAF, global assessment of functioning.

**Table 2:** Estimated coefficients (*P*-values) of log-transformed activity counts averaged within one of the five time intervals by diagnostic subgroups adjusted for the effects of age, gender, weekend, medication and BMI

	*Time of day* *(hours)*				
	*00*:*00–06*:*00*	*06*:*00–12*:*00*	*12*:*00–16*:*00*	*16*:*00–20*:*00*	*20*:*00–00*:*00*
*Covariates*					
Age	**−0.018 (0.00)**	0.005 (0.14)	**−0.009 (0.00)**	**−0.011 (0.00)**	**−0.024 (0.00)**
Gender	**−**0.292 (0.06)	0.036 (0.76)	**0.168 (0.04)**	**0.181 (0.02)**	0.12 (0.27)
Weekend	**−0.242 (0.00)**	**−0.61 (0.00)**	0.017 (0.59)	**−0.065 (0.01)**	0.052 (0.14)
Medication	**−**0.022 (0.92)	**−**0.147 (0.36)	**−**0.161 (0.18)	**−**0.156 (0.19)	0.016 (0.91)
BMI	**0.033 (0.002)**	**−**0.009 (0.21)	**−0.013 (0.01)**	**−0.015 (0.003)**	0.005 (0.36)
					
*Mood disorder subgroups*					
Bipolar I	**−**0.043 (0.87)	**−**0.103 (0.61)	**−0.359 (0.02)**	**−0.384 (0.005)**	**−0.51 (0.007)**
Bipolar II	0.276 (0.27)	**−**0.042 (0.81)	**−**0.02 (0.86)	**−**0.038 (0.74)	0.213 (0.20)
Major depression	0.003 (0.99)	**−**0.161 (0.29)	**−**0.139 (0.21)	**−**0.114 (0.28)	**−**0.134 (0.35)

Abbreviation: BMI, body mass index. Bolded values indicate significant results at 95% confidence level.

**Table 3:** Estimated coefficients (*P*-values) of day-to-day variability for the average log activity counts by diagnostic subgroups and covariates compared with controls by time of day

	*Coefficients by time of day* *(hours)*				
	*00*:*00–06*:*00*	*06*:*00–12*:*00*	*12*:*00–16*:*00*	*16*:*00–20*:*00*	*20*:*00–00*:*00*
*Covariates*					
Age	0.001 (0.73)	0.002 (0.17)	0.001 (0.23)	0.002 (0.07)	−0.001 (0.43)
Gender	0.073 (0.29)	−0.076 (0.19)	**−0.095** (**0.03)**	−0.064 (0.08)	0.046 (0.30)
Medication	0.006 (0.95)	0.065 (0.42)	0.01 (0.87)	0.043 (0.40)	−0.086 (0.16)
BMI	−0.002 (0.61)	0 (0.95)	0.002 (0.46)	0.001 (0.75)	0.002 (0.46)
					
*Mood disorder subgroups*					
Bipolar I	−0.008 (0.95)	0.073 (0.46)	**0.169** (**0.02)**	0.023 (0.71)	−0.043 (0.57)
Bipolar II	**0.282** (**0.01)**	0.099 (0.30)	0.093 (0.19)	−0.029 (0.64)	−0.096 (0.19)
Major Depression	0.175 (0.07)	−0.079 (0.33)	−0.05 (0.41)	−0.048 (0.35)	0.09 (0.41)

Abbreviation: BMI, body mass index.

Control group is treated as the reference level. Bolded values indicate significant results at 95% confidence level.
